# Daily patterns of physical activity, sedentary behavior, and prevalent and incident depression—The Maastricht Study

**DOI:** 10.1111/sms.14235

**Published:** 2022-09-27

**Authors:** Vincenza Gianfredi, Nicolaas C. Schaper, Anna Odone, Carlo Signorelli, Andrea Amerio, Simone J. P. M. Eussen, Sebastian Köhler, Hans H. C. M. Savelberg, Coen D. A. Stehouwer, Pieter C. Dagnelie, Ronald M. A. Henry, Carla J. H. van der Kallen, Marleen M. J. van Greevenbroek, Miranda T. Schram, Annemarie Koster

**Affiliations:** ^1^ CAPHRI Care and Public Health Research Institute Maastricht University Maastricht The Netherlands; ^2^ CARIM School for Cardiovascular Diseases Maastricht University Maastricht The Netherlands; ^3^ Department of Biomedical Sciences for Health University of Milan Milan Italy; ^4^ Department of Internal Medicine Maastricht University Maastricht The Netherlands; ^5^ Department Public Health, Experimental and Forensic Medicine University of Pavia Pavia Italy; ^6^ Vita‐Salute San Raffaele University Milan Italy; ^7^ Department of Neuroscience, Rehabilitation, Ophthalmology, Genetics, Maternal and Child Health (DINOGMI), Section of Psychiatry University of Genoa Genoa Italy; ^8^ IRCCS Ospedale Policlinico San Martino Genoa Italy; ^9^ Mood Disorders Program Tufts Medical Center Boston Massachusetts USA; ^10^ Department of Epidemiology Maastricht University Maastricht The Netherlands; ^11^ MHeNS School for Mental Health and Neuroscience Maastricht University Maastricht The Netherlands; ^12^ Department of Psychiatry and Neuropsychology Maastricht University Maastricht The Netherlands; ^13^ Department of Nutrition and Movement Sciences Maastricht University Maastricht The Netherlands; ^14^ School of Health Professions Education Maastricht University Maastricht The Netherlands; ^15^ NUTRIM, School for Nutrition and Translation Research Maastricht Maastricht University Maastricht The Netherlands; ^16^ Heart and Vascular Center Maastricht University Medical Center+ Maastricht The Netherlands; ^17^ Department of Social Medicine Maastricht University Maastricht The Netherlands

**Keywords:** 24‐hour distribution, depression, objectively measured physical activity, prospective study, sedentary behavior

## Abstract

This study aims to compare the accelerometer‐measured daily patterns of PA and sedentary behavior among participants with and without prevalent/incident depressive symptoms. We used data from 5582 individuals in The Maastricht Study (59.9 ± 8.6 years, 50.3% women). Daily patterns of sedentary time, light‐intensity physical activity (LiPA), moderate‐to‐vigorous physical activity (MVPA), and sit‐to‐stand transitions were objectively measured at baseline with the activPAL3 activity monitor. Depressive symptoms were assessed using the 9‐item Patient Health Questionnaire, both at baseline and annually (median follow‐up: 5.1 years). General linear models were used to compare patterns of physical activity and sedentary behavior between those with and without prevalent/incident depressive symptoms. Participants with prevalent depressive symptoms had significantly more sedentary time (18.6 min/day) and lower LiPA (26.8 min/day) and MVPA (4.8 min/day) than participants without depressive symptoms. Considering the daily patterns, participants with prevalent depressive symptoms had significantly more sedentary time early in the afternoon (12:00–18:00), early evening (18:00–21:00), and during the night (00:00–03:00), less time in LiPA in all periods between 09:00–21.00 and less MVPA in the morning (09:00:12:00), early afternoon (12:00–15:00), and evening (18:00–21:00), than those without. Similar differences in activity and sedentary behavior patterns between those and without incident depressive symptoms were observed albeit the differences were smaller. Overall, we did not find specific time slots particularly associated with both prevalent and incident depressive symptoms. These findings may indicate that less sedentary time and more intense PA can be important targets for the prevention of depression irrespective of the timing of the day.

## 1 INTRODUCTION

Depression is one of the most important health problems globally, being among the first 20 leading causes of disability.^1^ Approximately 350 million people are affected worldwide, accounting for 4.4% of the population, and by 2030, it is expected to become the leading cause of disease in the world.^1^ Depression has a high psychosocial impact demanding targeted psychological and pharmacological support. However, available therapies are not highly effective, with a large group of patients having therapy‐resistant depression or relapses, which highlights the need for other treatment options and more effective preventive strategies. In this regard, physical activity (PA) is known to be beneficial for mental health and to reduce depressive symptoms. The underlying scientific evidence of the World Health Organization (WHO) guidelines on PA shows the importance of participating in moderate‐to‐vigorous physical activity (MVPA) for mental health,^2^ and well‐established evidence suggests that increasing PA can reduce the risk of depression.^3^ The WHO physical activity guidelines also acknowledge the role of more light‐intensity physical activity (LiPA). Conversely, more recent evidence shows that higher levels of sedentary behavior, especially in combination with mentally passive behaviors, such as screen time, might be associated with higher risk of depression.^4^

Even though growing evidence suggests the (biological) plausibility of the beneficial effects of PA on depression and depressive symptoms,^5^, ^6^, ^7^ little is known about how daily patterns of PA and sedentary behavior are associated with depression. Most of the current evidence is based on self‐reported measurements of PA^3^—which may be influenced by individual mood, memory inaccuracy, and social desirability bias—while the availability of accelerometers allows a more detailed objective assessment of daily PA. Evaluating the association between daily patterns (level and timing) of PA and sedentary time with depression is important to identify potential differences in the distribution of PA and sedentary time over the day responsible for the higher risk of developing depression. Further, this potential different distribution of PA and sedentary behavior among individuals with and without depression may provide tools for tailored interventions that use optimized timing of sedentary/active time during the day.

The aim of this study is to compare accelerometer‐measured daily patterns of PA and sedentary behavior among participants with and without prevalent and incident depressive symptoms in The Maastricht Study, a large population‐based cohort study.

## 2 MATERIALS AND METHODS

### 2.1 Study population and design

**FIGURE 1:**
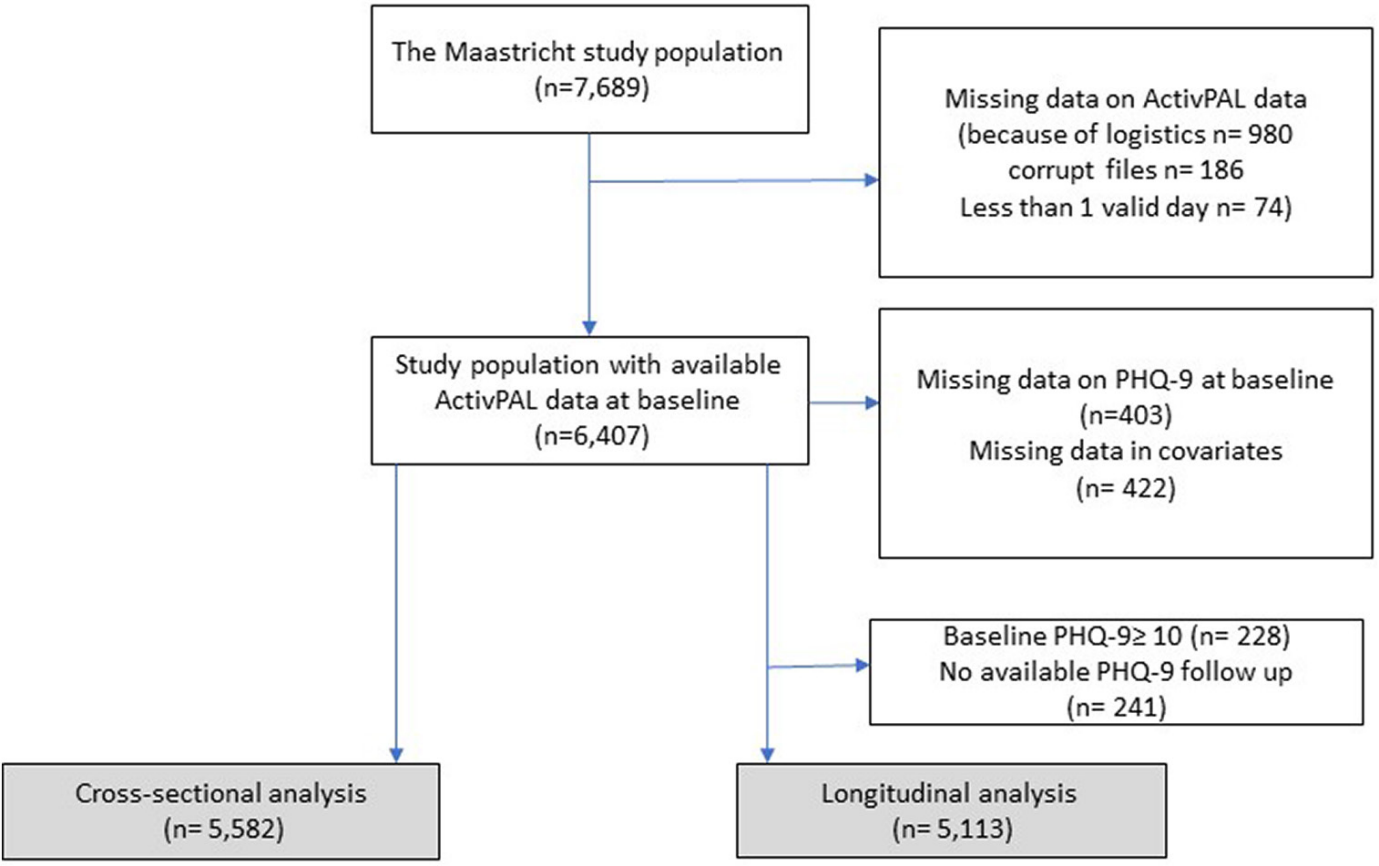
Flowchart of the study population.

We used data from The Maastricht Study, a population‐based observational prospective cohort study. A detailed rationale and methodology were published elsewhere.^8^ In brief, the study focuses on the etiology, pathophysiology, complications, and comorbidities of type 2 diabetes mellitus (T2DM) and is characterized by an extensive phenotyping approach. Eligible individuals were all individuals aged between 40 and 75 years and living in the southern part of the Netherlands. Individuals were recruited through mass media campaigns and from the municipal registries and the regional Diabetes Patient Registry via mailings. Recruitment was stratified according to known T2DM status, with an oversampling of individuals with T2DM, for reasons of efficacy. The present analysis includes data from the first 7689 individuals, who completed the baseline measurements between November 2010 and December 2017. The baseline examinations of each participant were performed within 3 months, and follow‐up surveys, via questionnaires, were collected annually after the first examination. Annual follow‐up data were available in 90.8% (year 1), 83.2% (year 2), 77.8% (year 3), 68.6% (year 4), 58.8% (year 5), 35.3% (year 6), and 16.0% (year 7) of the individuals. The relatively low number of individuals for years 6 and 7 is because the follow‐up measurement is still ongoing. For the present study, we used complete cases, so individuals were excluded from the analysis when they did not receive an accelerometer due to logistical problems (*n* = 1282), no baseline data were available on depressive symptoms (*n* = 403), individuals had missing data for covariates (*n* = 422). The final sample size for the analyses with prevalent depressive symptoms was *n* = 5582. For the analyses with incident depressive symptoms, 228 individuals with depressive symptoms at baseline (9‐item Patient Health Questionnaire; PHQ‐9 ≥ 10) and with missing data for follow‐up depressive symptoms (*n* = 241) were also excluded, resulting in a final sample size of *n* = 5113 individuals. Figure 1 shows the flowchart of the study population.

The Institutional Medical Ethics Committee (NL31329.068.10) and Ministry of Health, Welfare and Sports of the Netherlands (Permit 131 088‐105 234‐PG) ethically approved the study. Written informed consent was required to all individuals.

### 2.2 Physical activity and sedentary behavior measurements

Physical activity and sedentary behavior were measured at baseline using the activPAL3™ physical activity monitor (PAL Technologies), a small (53 × 53 × 7 mm) and light‐weight (15 g) triaxial accelerometer that records movement in the vertical, anteroposterior, and medio‐lateral axes, and also determine posture (sitting, lying, standing, and stepping) based on acceleration information. The device was attached directly to the skin on the front of the right thigh with transparent 3 M Tegaderm™ tape, after the device had been waterproofed with a nitrile sleeve. Individuals were asked to wear the accelerometer for 8 consecutive days, without removing it at any time, and in case this happened, instructions not to replace it were provided. Data from the first day were excluded from the analysis because individuals performed physical function tests at the research center after the device was attached. In addition, data from the final wear day providing ≤14 waking hours of data were excluded from the analysis.^9^ Individuals were included if they provided at least 1 valid day (≥10 h of waking data). Data were uploaded using the activPAL software and processed using software developed in MATLAB® 2018b (MathWorks).^9^

Variables used in this analysis were average minutes of sedentary time during wake time, light intensity physical activity (LiPA), and moderate‐to‐vigorous physical activity (MVPA). LiPA was calculated as the sum of standing minutes and of minutes with a step frequency < 100 steps/minute.^10^ MVPA was calculated as the minutes with a step frequency ≥ 100 steps/min. Sit‐to‐stand transitions were defined as transitions in posture from sitting/lying to standing. The method used to estimate waking time has been published previously.^9^ In addition to total average time (min) in LiPA, MVPA, sedentary time, and average number of sit‐to‐stand transition per day, all the estimated variables were calculated as minutes of sedentary time, LiPA, MVPA, and number of sit‐to‐stand transitions for each hour and for 3‐h time slots for an average 24‐h day and separately for week and weekend days.

### 2.3 Assessment of depression at baseline

At baseline, severity and presence of depressive symptoms were assessed by means of a validated Dutch version of the 9‐item Patient Health Questionnaire (PHQ‐9).^11^ The PHQ‐9 is a self‐administered questionnaire based on the DSM‐IV criteria for major depressive disorder (MDD). The PHQ‐9 measures both cognitive symptoms of depression and somatic symptoms of depression.^12^ Each item is rated on a 4‐point ordinal scale from 0 = “not at all” to 3 = “nearly all of the time.” The total score ranges from 0 to 27. When one or two items were missing, the total score was calculated as 9 × (total points/ [9 − number of missing items]) and rounded to the nearest integer. A pre‐defined cut‐off score of ≥10 was used as a dichotomous scoring system for defining clinically relevant depressive symptoms.^13^

In addition to depressive symptoms, prevalent and lifetime major depressive disorder (MDD) was assessed by the Mini‐International Neuropsychiatric Interview (MINI) at baseline.^14^ The MINI is a short diagnostic structured interview, used to assess the presence of minor or major depressive disorder in the preceding 2 weeks according to the DSM‐IV (Diagnostic and Statistical Manual of Mental Disorders, Fourth Edition). MDD was diagnosed if an individual had at least one core symptom (i.e., depressed mood or loss of interest) and at least four other symptoms of depression (i.e., significant weight change of change in appetite, insomnia or hypersomnia, psychomotor agitation or retardation, fatigue or loss of energy, guilt or worthlessness, diminished ability to think or concentrate or indecisiveness, and suicidal thoughts or plans). Lifetime history of MDD was assessed by asking for the presence of symptoms during minimally 2 weeks in lifetime.

### 2.4 Assessment of depressive symptoms during follow‐up

Incident clinically relevant depressive symptoms were assessed by use of the PHQ‐9 questionnaire annually during 7 years of follow‐up. Incident clinically relevant depressive symptoms were defined as no depressive symptoms at baseline (PHQ‐9 < 10) and presence of depressive symptoms (PHQ‐9 ≥ 10) on at least one follow‐up moment.

### 2.5 Covariates

Covariates included sex, age, level of education, BMI, lifestyle factors (smoking status, alcohol consumption, energy intake), and biomedical parameters (diabetes status, use of antidepressant drugs, mobility limitation, hypertension, total cholesterol‐to‐HDL cholesterol ratio, and history of cardiovascular diseases [CVD]). Level of education was divided in three categories: low = no education/primary education/lower vocational education, medium = intermediate vocational education/higher secondary education/higher vocational education, high = higher professional education/university education. BMI was calculated from weight and height measured in a physical examination to the nearest of 0.5 kg or 0.1 cm. Smoking status was divided in three categories: current, former, and never smokers. Alcohol consumption was divided into three categories: non‐consumers, low consumers (for women ≤7 glasses alcoholic beverages per week; for men ≤14 glasses alcoholic beverages per week), and high consumers (for women >7 glasses per week; for men >14 glasses per week).^15^ Energy intake was expressed in kcal/day and measured by a validated Food Frequency Questionnaire.^16^ Individuals with implausible energy intake (men: energy intake (kcal) <800 kcal or >4200 kcal; women: energy intake (kcal) <500 kcal or >3500 kcal) were excluded (*n* = 140).^17^ Type 2 diabetes status was defined by a standardized 2‐h 75‐g oral glucose tolerance test after an overnight fast and use of antidiabetic medication as previously described.^8^ Antidepressant drug use was assessed in a medication interview where generic name, dose, and frequency were registered. Mobility limitation was acquired from the Dutch version of the Short Form Health Survey and was defined as having difficulty with stair climbing and/or walking 500 m. Office blood pressure, plasma glucose levels, and plasma lipid profile were measured as described before.^8^ CVD history was derived from the Rose questionnaire and defined as a self‐reported history of any of the following conditions: myocardial infarction, cerebrovascular infarction or hemorrhage, percutaneous artery angioplasty of, or vascular surgery on, the coronary, abdominal, peripheral, or carotid arteries.

### 2.6 Statistical analysis

Descriptive characteristics of the study population are presented as mean and standard deviation (SD) for continuous variables or numbers and percentages for categorical variables. To assess differences between individuals with and without depressive symptoms, we performed chi‐square and analysis of variance (ANOVA) tests where appropriate. General linear models were used to compare daily patterns of sedentary time and LiPA, MVPA, and sit‐to‐stand transitions (dependent variables) between participant with and without prevalent/incident depressive symptoms (independent variables). We tested for difference per hour and using 3‐h time slots starting from midnight. In the main analyses, we present the differences for the 3‐h time slots for the ease of interpretation and to reduce the number of statistical tests performed. We adjusted for several covariates in the analyses: sociodemographic factors (age, sex, level of education) and, because of the oversampling, type 2 diabetes. Moreover, we adjusted for smoking status, alcohol consumption, energy intake, BMI, hypertension, total cholesterol‐to‐HDL cholesterol ratio, history of CVD. Sensitivity analyses encompassed further adjustment for (1) mobility limitation, (2) MVPA (only performed for LiPA and sedentary time), (3) occupational status, (4) antidepressant drug use, (5) MDD at baseline; in addition, we excluded individuals (6) that used antidepressant drugs at baseline, and only for the analysis with incident depressive symptoms (7) individuals with MDD at baseline, and (8) individuals with lifetime MDD. Moreover, we restricted the analysis to (9) a group with maximum 2 missing follow‐up measurements for the analyses with incident depressive symptoms. Lastly, we tested the interactions between sex and prevalent/incident depressive symptoms for the 3‐h time slots of all the estimated variables (sedentary time, LiPA, MVPA, and number of sit‐to‐stand transitions) in the general linear models. Similarly, interactions with diabetes status were tested. All analyses were conducted using IBM SPSS software version 21.0 (IBM Corp). Associations with *p* < 0.05 in two‐sided tests were considered to be statistically significant.

## 3 RESULTS

### 3.1 Descriptive characteristics of the population

Table 1 shows the characteristics of the population at baseline (*n* = 5582) and the population with follow‐up data (*n* = 5113) stratified by presence of prevalent (yes/no) and incident (yes/no) depressive symptoms, respectively. The mean age of the baseline cohort was 59.9 ± 8.6 years, and 50.3% were women. On average, individuals with prevalent or incident depressive symptoms had a lower level of education, were more often smokers, had a higher BMI, were less physically active, and had a less favorable cardiovascular‐risk profile compared to individuals without depressive symptoms. Comparing individuals with full data (included in the current analyses) and those with missing data (excluded from the analyses), individuals with missing data had more depressive symptoms and a worse cardiovascular‐risk profile compared to the included individuals (Table S1).

**TABLE 1:** Characteristics of the study population stratified according to prevalent depressive symptoms (yes/no) and incident depressive symptoms (yes/no)

Characteristic	Total population at baseline (*n* = 5582)	No prevalent depressive symptoms (*n* = 5354)	Prevalent depressive symptoms (*n* = 228)	*p*‐value	Total population with follow‐up data (*n* = 5113)	No incident depressive symptoms (*n* = 4549)	Incident depressive symptoms (*n* = 564)	*p*‐value
Sex (women), *n* (%)	2809 (50.3)	2674 (49.9)	135 (59.2)	0.006	2559 (50.0)	2256 (49.6)	303 (53.7)	0.064
Age (years)	59.9 ± 8.6	60.1 ± 8.6	56.5 ± 8.8	<0.001	60.1 ± 8.5	60.1 ± 8.5	59.9 ± 8.5	0.590
Education, *n* (%)								
Low	1891 (33.9)	1793 (33.5)	98 (43.0)	<0.001	1682 (32.9)	1455 (32.0)	227 (40.2)	<0.001
Medium	1537 (27.5)	1467 (27.4)	70 (30.7)		1408 (27.5)	1253 (27.5)	155 (27.5)	
High	2154 (38.6)	2094 (39.1)	60 (26.3)		2023 (39.6)	1841 (40.5)	182 (32.3)	
Smoking, *n* (%)				<0.001				<0.001
Never	2115 (37.9)	2048 (38.3)	67 (29.4)		1962 (38.4)	1783 (39.2)	179 (31.7)	
Former	2793 (50.0)	2684 (50.1)	109 (47.8)		2573 (50.3)	2286 (50.3)	287 (50.9)	
Current	674 (12.1)	622 (11.6)	52 (22.8)		578 (11.3)	480 (10.6)	98 (17.4)	
Alcohol consumption, *n* (%)				<0.001				<0.001
None	981 (17.6)	899 (16.8)	82 (36.0)		835 (16.3)	696 (15.3)	139 (24.6)	
Low	3304 (59.2)	3.196 (59.7)	108 (47.4)		3052 (59.7)	2742 (60.3)	310 (55.0)	
High	1297 (23.2)	1.259 (23.5)	38 (16.7)		1226 (24.0)	1111 (24.4)	115 (20.4)	
Having a partner (yes), *n* (%)	4707 (84.4)	4.555 (85.1)	152 (66.7)	<0.001	4.364 (85.4)	3.917 (86.2)	447 (79.3)	<0.001
Energy intake (Kcal)	2128.3 ± 592.1	2127.9 ± 590.8	2138.4 ± 622.5	0.793	2126.4 ± 586.2	2124.7 ± 583.2	2140.1 ± 609.6	0.558
Body mass index (kg/m^2^)	26.9 ± 4.4	26.8 ± 4.3	28.5 ± 5.8	<0.001	26.8 ± 4.3	26.6 ± 4.1	27.9 ± 5.2	<0.001
Hypertension, *n* (%)	2989 (53.5)	2854 (53.3)	135 (59.2)	0.080	2721 (53.2)	2381 (52.3)	340 (60.3)	<0.001
Total cholesterol‐to‐HDL cholesterol ratio, *n* (%)	3.6 ± 1.2	3.6 ± 1.2	3.8 ± 1.5	<0.001	3.6 ± 1.1	3.5 ± 1.1	3.7 ± 1.2	0.026
History of CVD, *n* (%)	928 (16.6)	875 (16.3)	53 (23.2)	0.006	828 (16.2)	715 (15.7)	113 (20.0)	0.009
Type 2 diabetes, *n* (%)	1298 (23.3)	1221 (22.8)	77 (33.8)	<0.001	1136 (22.2)	956 (21.0)	180 (31.9)	<0.001
Depression								
Depression score at baseline (PHQ‐9 score)	2.7 ± 3.3	3.6 ± 1.2	13.6 ± 3.8	<0.001	2.29 ± 2.3	2.0 ± 2.1	4.5 ± 2.7	<0.001
Major depressive disorder at baseline (MINI), *n* (%)	152 (2.8)	75 (1.5)	77 (35.2)	<0.001	68 (1.4)	42 (1.0)	26 (4.8)	<0.001
Major depressive disorder lifetime (MINI), *n* (%)	1635 (30.4)	1449 (28.1)	186 (84.5)	<0.001	1381 (28.1)	1091 (24.9)	290 (54.1)	<0.001
Use of antidepressants at baseline	361 (6.5)	295 (5.5)	66 (28.9)	<0.001	282 (5.5)	204 (4.5)	78 (13.8)	<0.001
Physicaly activity								
Sedentary (min/day)	561.1 ± 100.6	560.3 ± 99.7	581.0 ± 119.3	0.002	559.9 ± 99.1	558.5 ± 98.8	571.0 ± 100.8	0.005
LiPA (min/day)	343.5 ± 94.6	344.7 ± 93.9	316.5 ± 106.6	<0.001	344.9 ± 93.3	346.6 ± 93.0	331.6 ± 94.8	<0.001
MVPA (min/day)	40.1 ± 23.1	40.5 ± 23.1	32.6 ± 21.4	<0.001	40.7 ± 23.2	41.3 ± 23.1	36.0 ± 23.5	<0.001
Transitions (*n*/day)	54.6 ± 14.3	54.6 ± 14.2	53.6 ± 16.4	0.277	54.7 ± 14.1	54.7 ± 14.0	54.2 ± 14.8	0.400

*Note*: Results are presented as mean ± standard deviation (SD) or *n* (%).

Abbreviations: CVD, Cardiovascular diseases; LiPA, Light physical activity; MDD, Major Depressive Disorder; MINI, Mini‐International Neuropsychiatric Interview; MVPA, Moderate‐to‐vigorous physical activity; PHQ‐9, 9‐item Patient Health Questionnaire.

### 3.2 Daily patterns of sedentary behavior and PA according to prevalent depressive symptoms

**FIGURE 2:**
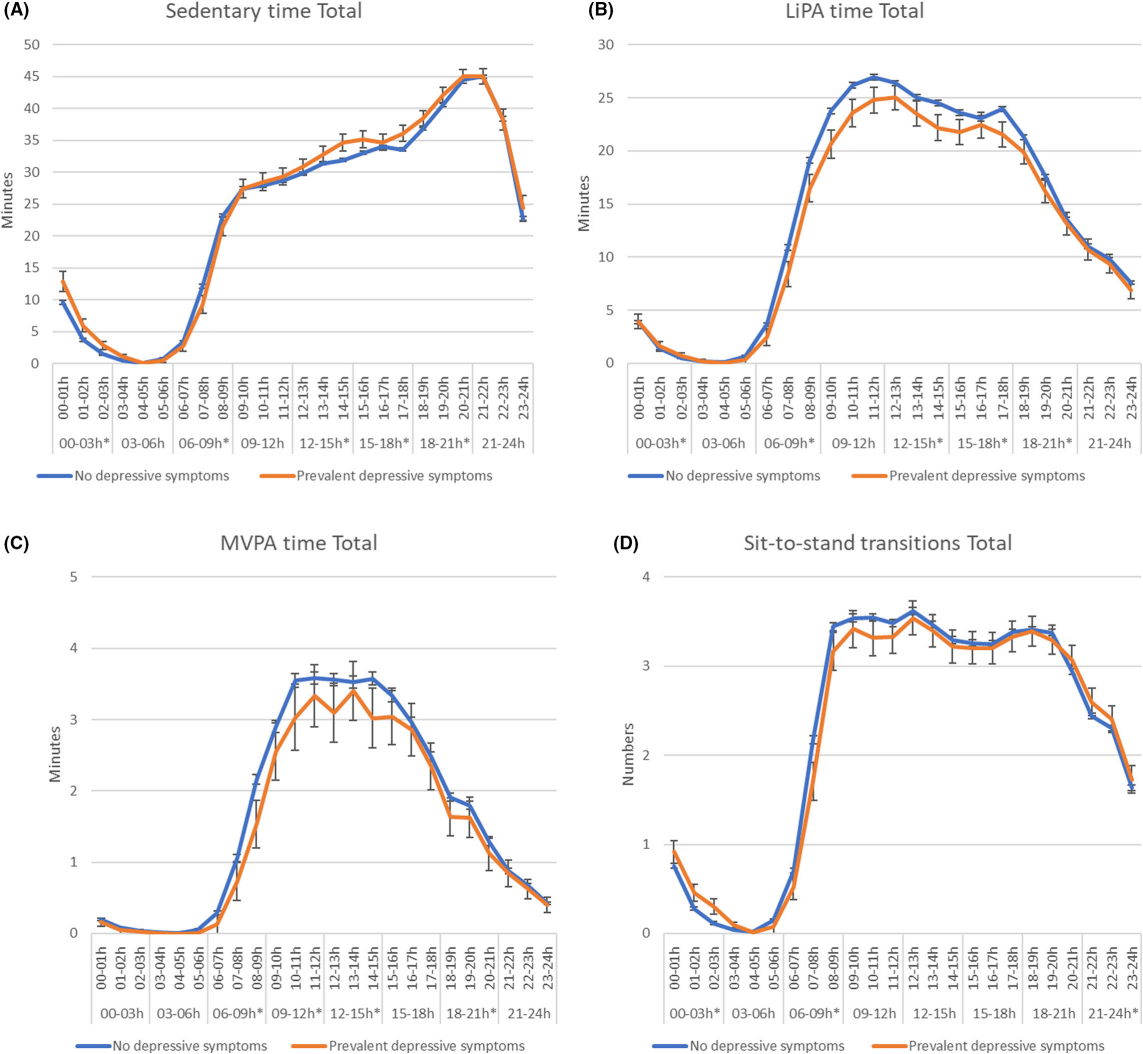
Hourly distribution of (A) sedentary time, (B) LiPA, (C) MVPA, and (D) sit‐to‐stand transitions in individuals with and without prevalent depressive symptoms. Statistically significant differences in time slots are reported with*. Model 3: adjusted for age, sex, level of education, type 2 diabetes, smoking status, alcohol consumption, energy intake, BMI, hypertension, cholesterol, history of CVD.

Participant with prevalent depressive symptoms had significant higher total daily sedentary time: mean sedentary time (95% CI): 579.0 min (566.7–591.4) versus 560.4 min (557.9–562.9) in those without prevalent depressive symptoms (*p* = 0.004, Table S2). When analyzing different time slots (Figure 2A and Table S2), individuals with prevalent depressive symptoms had statistically significantly more sedentary time (all *p* < 0.05) in the afternoon (12:00–15:00: 4.8 min, and 15:00–18:00: 5.1 min), early evening (18:00–21:00: 3.8 min), and during the night (00:00–03:00: 6 min) compared to those without prevalent depressive symptoms. In contrast, individuals with prevalent depressive symptoms had statistically significant less sedentary time early in the morning (06:00–09:00: 4.7 min) compared to those without these symptoms. Similar differences were observed when we examined patterns of physical activity and sedentary behavior separately on weekday and weekend days (Figure S1).

In the adjusted model, total daily LiPA was lower in the participants with prevalent depressive symptoms compared to those without prevalent depressive symptoms, 317.8 min (305.8–329.8) versus 344.6 min (342.1–347.0) (*p* < 0.001, Table S2). As shown in Figure 2B (and Table S2), individuals with prevalent depressive symptoms had statistically significantly less LiPA in all timeslots of the day: (6:00–9:00: 5.7 min, 9:00–12:00: 7.3 min, 12:00–15:00: 4.8 min, 15:00–18:00: 4.7 min, and 18:00–21:00: 3.1 min less LiPA) compared to those without these symptoms. Similar associations of prevalent depressive symptoms with LiPA were observed for weekday and weekend days (Figure S1).

In addition to LiPA, also total daily MVPA time was lower in individuals with depressive symptoms compared to those without prevalent depressive symptoms in the adjusted model, 35.5 min (32.7–38.4) versus 40.3 min (39.7–40.9), (Table S2, *p* = 0.001). As shown in Figure 2C (and in Table S2), individuals with prevalent depressive symptoms had statistically significantly lower levels of MVPA during the morning and early in the afternoon (06:00–09:00: 1.1 min, 09:00–12:00: 1.1 min, and 12:00–15:00: 1.2 min less MVPA) and during the evening (18:00–21:00: 0.7 min less MVPA) compared to those without prevalent depressive symptoms. When considering weekdays and weekend days separately, the associations of prevalent depressive symptoms with MVPA were most pronounced during weekend days (Figure S1).

Finally, as shown in Figure 2D (and in Table S2), total daily sit‐to‐stand transitions were comparable in the individuals with and without prevalent depressive symptoms in the adjusted model, 53.8 (52.0–55.7) versus 54.6 (54.2–55.0), *p* = 0.430. Individuals with prevalent depressive symptoms had statistically fewer transitions during the morning (6:00–9:00: 0.9 less transitions) and more transitions during in‐bed time (21:00–03:00: 1.0 more transitions) compared to those without prevalent depressive symptoms. When considering weekdays and weekend days separately, no differences were found (Figure S1).

### 3.3 Daily patterns of sedentary behavior and PA according to incident depressive symptoms

**FIGURE 3:**
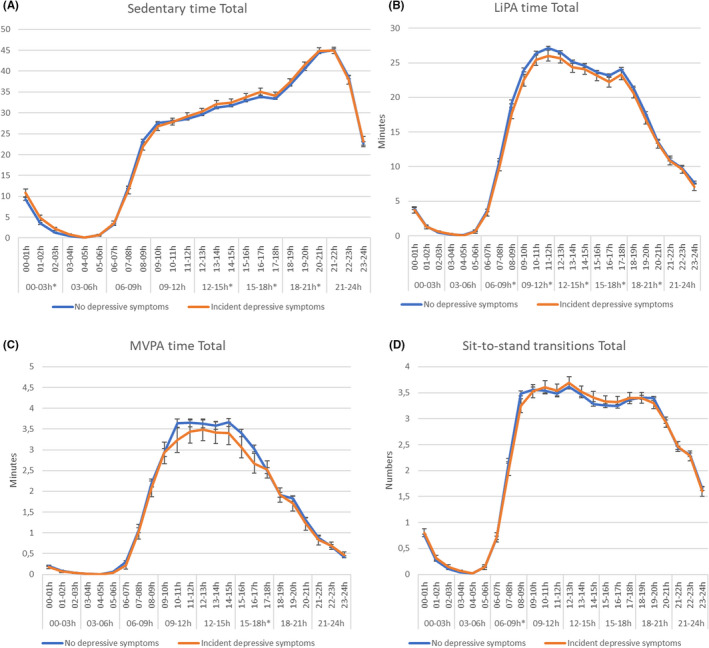
Hourly distribution of (A) sedentary time, (B) LiPA, (C) MVPA, and (D) sit‐to‐stand transitions in individuals with and without incident depressive symptoms. Statistically significant differences in time slots are reported with*. Model 3: adjusted for age, sex, level of education, type 2 diabetes, smoking status, alcohol consumption, energy intake, body mass index, hypertension, cholesterol, history of cardiovascular diseases.

Figure 3A–D shows the hourly patterns of sedentary behavior and PA over 24 h by incident depressive symptoms in the adjusted model, over a median follow‐up of 5.1 years.

Total daily sedentary time was in the adjusted model higher in individuals with incident depressive symptoms compared to those without depressive symptoms, 567.3 min (559.7–575.0) versus 559.1 min (556.4–561.8) (Table S3, *p* = 0.046). As shown in Figure 3A (and Table S3), individuals with incident depressive symptoms had statistically more sedentary time during early (12:00–15:00: 2.9 min) and late afternoon (15:00–18:00: 4.1 min), early evening (18:00–21:00: 3.2 min) and during the night (00:00–03:00: 4.3 min more sedentary time) compared to those without incident depressive symptoms. When considering weekdays and weekend days separately (Table S3), individuals with incident depressive symptoms, compared to those without, had higher daily sedentary time (adjusted model) during the week and during the weekend, 565.4 min (556.1–574.8) versus 553.2 min (549.9–556.5), *p* = 0.015 and (559.9–576.6) versus 562.0 min (559.0–564.9), *p* = 0.164, compared to those without these symptoms, but the difference in sedentary time seemed more pronounced during the weekend days.

Total daily LiPA was in the adjusted model lower in participants with incident depressive symptoms compared to those without depressive symptoms, 332.8 min (325.3–340.2) versus 346.3 min (343.7–348.9) (Table S3, *p* = 0.001). As shown in Figure 3B (and Table S3), individuals with incident depressive symptoms had less LiPA during almost all day‐time timeslots (6:00–9:00: 2.7 min 9:00–12:00: 3.0 min, 12:00–15:00: 2.0 min, 15:00–18:00: 2.6 min and 18:00–21:00: 2.8 min less LiPA) compared to those without. No difference was observed in the evening/night (between 21:00 and 06:00 h). Similar patterns were observed for weekdays and weekend days (Figure 2B and Table S3).

In addition to LiPA, also total daily MVPA was in adjusted model lower in participants with incident depressive symptoms compared to those without incident depressive symptoms, 38.6 min (36.8–40.4) versus 40.9 min (40.3–41.5), *p* = 0.016. As shown in Figure 3C (and Table S3), individuals with incident depressive symptoms had lower MVPA in the afternoon (15:00–18:00: 0.6 min less MVPA) compared to those without incident depressive symptoms. When considering weekdays and weekend days separately, the difference between individuals with and without incident depressive symptoms became more pronounced during weekend days, in particular during 12:00 and 18:00 (Table S3 and Figure 2C).

Regarding the sit‐to‐stand transitions, total daily transitions in the adjusted model were similar in participants with incident depressive symptoms compared to those without depressive symptoms, 54.7 (53.6–55.8) versus 54.6 (54.2–55.0) (Table S3, *p* = 0.840). Also, the pattern over the day was similar for those with and without incident depressive symptoms with only a small statistically significant difference observed during the early morning (6:00–9:00, with 0.4 fewer transitions in those with incident depressive symptoms), as shown in Figure 3D (and Table S3). Similar patterns were observed for weekdays and weekend days (Figure 2D and Table S3).

### 3.4 Additional analyses

Sensitivity analyses revealed similar associations after (1) additional adjustment for mobility limitation, (2) additional adjustment for MVPA in the sedentary time and LiPA analyses, (3) additional adjustment for occupational status, (4) additional adjustment for antidepressant drug use, (5) additional adjustment for MDD at baseline, (6) excluding individuals with antidepressant drug use at baseline, and only for the analysis with incident depressive symptoms (7) excluding individuals with MDD at baseline, (8) excluding individuals with lifetime MDD, and (9) admitting at maximum two missing follow‐up measurements (only for the analyses with incident depressive symptoms). The differences in the daily pattern of sedentary behavior and physical activity between individuals with and without prevalent depressive symptoms and the daily pattern of sedentary behavior and physical activity did not materially change the results. In sensitivity analyses, the differences between individuals with and without incident depressive symptoms were similar, but became slightly weaker for the analyses after excluding individuals with MDD at baseline (Table S4) or excluding individuals with antidepressant drug use at baseline (Table S5). Interactions between prevalent/incident depressive symptoms with sex and diabetes status for the 3‐h time slots of all the estimated variables (sedentary time, LiPA, MVPA, and number of sit‐to‐stand transition) were tested and were not statistically significant (*p* > 0.10).

## 4 DISCUSSION

We evaluated the daily patterns of objectively measured sedentary behavior and PA in people with prevalent and incident depressive symptoms in a population‐based setting. Our study shows that people with prevalent depressive symptoms had more sedentary time during wake‐time, especially during afternoon, early evening, and night time, than people without these symptoms, with similar results for weekdays and weekend days. Moreover, individuals with prevalent depressive symptoms had statistically less LiPA as well as less MVPA throughout the day, fewer sit‐to‐stand transitions during daytime hours, and more transitions during the night (that could also be during in‐bed time) compared to those without prevalent depressive symptoms. Similar results were observed in participants with incident depressive symptoms compared to those without, albeit the differences were smaller. Individuals with incident depressive symptoms had more sedentary time, especially during the afternoon and evening hours, and particularly on weekend days, compared to those without incident depressive symptoms. Further, participant with incident depressive symptoms had lower levels of LiPA throughout the whole day while only small differences in MVPA were observed. In particular, for MVPA, the difference between the two groups became more pronounced, during 12:00 and 18:00 when weekdays and weekend days were considered separately. This could be explained by the fact that participants without incident depressive symptoms engaged in more MVPA during the weekend compared to the rest of the week while those who developed these symptoms did not show higher MVPA during the weekend. Overall, participant with prevalent or incident depressive symptoms had lower levels of physical activity and spent more time sedentary throughout the day, without major differences in the pattern (level and timing) of physical activity and sedentary behavior between those with and without depressive symptoms.

Our study is, to the best of our knowledge, the first study comparing daily activity patterns, taking both level and timing into account by objectively measured PA and sedentary behavior, between participants with and without prevalent and incident depressive symptoms in a large cohort of adults living in the community. Only a few previous studies have assessed the association between daily activity pattern and prevalent depression.^18^, ^19^, ^20^ A small case–control study in 121 individuals (of which 58 with depressive symptoms) found that individuals with prevalent depressive symptoms were more active during the night compared to individuals without depressive symptoms but less active during the morning and early in the afternoon.^18^ Another study performed among dementia caregivers found that caregivers with depressive symptoms were less physically active only in the morning (from 8:00 to 10:00), compared to caregivers without depressive symptoms.^19^ Our results show that participants with prevalent depressive symptoms were more sedentary (especially during the afternoon and early evening) and had overall lower levels of PA during the day, prominently during the morning than individuals without depressive symptoms, which may reflect symptoms of fatigue, low quality of sleep and anhedonia, which are part of the depression phenotype.^21^ Hence, we cannot exclude the possibility of a bidirectional association between PA and depressive symptoms. Indeed, previous research has shown that as a consequence of depression, people might be more sedentary and less active.^22^ At the same time, previous studies have also found an important role of PA in treating depression.^23^ As for instance, evidence suggests that aerobic exercise is effective in reducing depressive symptoms in adults, particularly for those with more severe depressive symptom.^24^ At the same time, replacing sedentary time with LiPA and MVPA reduces the risk of depression by 1.3% and 12.5%, respectively.^25^

Comparing participants with and without incident depressive symptoms in the current study, similar results were found as for prevalent depressive symptoms, albeit smaller differences in sedentary time and PA were observed. Our results support a temporal association of both less LiPA and MVPA with the development of depressive symptoms, although we cannot exclude misclassification of depressive symptoms at baseline that might have resulted in a reduction of physical activity. We have previously shown in a smaller sample of the Maastricht Study that lower levels of total amount of daily LiPA in women were associated with a higher risk of incident depressive symptoms.^26^ Results from the current analyses suggest that lower levels of LiPA are associated with prevalent and incident depression in women as well as in men and that the total amount of LiPA is more important than the pattern in which activity is spread throughout the day. In light of this, future interventions may focus on reducing the total volume of sedentary time and increasing physical activity rather than concentrating on the activities in some specific hours of the day. More focus on low‐intensity activities throughout the day could be of interest as this might be relatively more feasible, effective, and cost‐effective.^27^, ^28^

### 4.1 Potential biological mechanisms

Several mechanisms may explain the association of PA, sedentary behavior, and depression. PA seems to play an important role in mood regulation by reducing the hypothalamic–pituitary–adrenal axis reactivity, which appears to be upregulated in depression due to chronic stressors.^29^ In vivo studies have shown a serotonergic activity of PA. Indeed, higher level of both noradrenalin and serotonin has been found after PA, suggesting that PA and serotoninergic antidepressant drug may simulate the same effects.^30^ Moreover, PA may increase brain‐derived neurotrophic factor levels, which are associated with both cognitive and emotional enhancement, especially after regular PA.^31^ PA seems also able to reduce the inflammatory status which is interrelated with improvement of vascular and microvascular function^32^; both, inflammation and microvascular dysfunction are implicated in the etiology of depression.^33^, ^34^, ^35^ In addition, in an earlier study in the same cohort, we observed that a lower level cardio‐respiratory fitness was associated with a higher risk of depressive symptoms, and PA is one of the modifiable factors able to improve cardio‐respiratory fitness.^36^ Cardio‐respiratory fitness reflects the capacity of the body in transporting and using oxygen and nutrients, and a reduced supply of oxygen and nutrients to the brain could contribute to poorer perfusion of areas involved in mood regulation.

### 4.2 Limitations and strengths

Some limitations of this study need to be taken into account when interpreting its findings. PA and sedentary behavior data were collected only at baseline, and potential changes over time could not be considered. Moreover, as reported in Table S1, individuals with depressive symptoms or with severe depression may be less likely to participate, leading to potential selection bias, which probably resulted in an underestimation of the strength of the associations. Lastly, the PHQ‐9 is not a diagnostic tool for MDD, an PHQ‐9 score higher than 10 is suggestive for depressive symptoms, but does not necessarily equate with MDD. However, it is a validated instrument, with high sensitivity and specificity,^14^ and cross‐sectional analyses with the MINI showed remarkably similar results, misclassifications are therefore expected to be low.^11^ Moreover, the comparison between PHQ‐9 and MDD diagnosis has been performed within the dataset of The Maastricht Study in a previous publication that found a good level of sensitivity and specificity.^37^

Important strengths of this study include the objectively measured physical behavior. Measurement of physical activity and sedentary behavior was performed with the activPAL activity monitor, allowing for an objective and continuous activity assessment in daily life over multiple days and the assessment of detailed daily patterns physical behavior. The measurements obtained by the thigh‐worn accelerometer is based on a combination of both tri‐axial acceleration and posture which has been proven to be more accurate in measuring sedentary time and transitions in posture compared to instruments using only acceleration technology.^38^ Other strengths include the large sample size (228 individuals with depressive symptoms at baseline and 564 with incident depressive symptoms during 7 years of follow‐up) and extensive data collection enabling to control for most important covariates.

## 5 PERSPECTIVE

Our data show that middle‐aged and older adults with prevalent depressive symptoms were more likely to be more sedentary and less physical active (of both light and moderate to vigorous intensity) during daytime compared to participants without depressive symptoms, even if no major differences in the hourly patterns over the day have been observed. The same trends, but with somewhat weaker associations, were observed with incident depressive symptoms. Overall, we did not find specific time slots particularly associated with both prevalent and incident depressive symptoms. These findings may indicate that less sedentary time and more intense PA can be important targets for the prevention of depression irrespective of the timing of the day.

## AUTHOR CONTRIBUTIONS

Authors VG, AK, and NCS designed the study. VG analyzed the data and wrote the first version of the manuscript. All authors read and critically revised the paper. All Authors approved the final version of the manuscript.

## FUNDING INFORMATION

This study was supported by the European Regional Development Fund via OP‐Zuid, the Province of Limburg, the Dutch Ministry of Economic Affairs (grant 31O.041), Stichting De Weijerhorst (Maastricht, The Netherlands), the Pearl String Initiative Diabetes (Amsterdam, The Netherlands), the Cardiovascular Center (CVC, Maastricht, the Netherlands), CARIM School for Cardiovascular Diseases (Maastricht, The Netherlands), CAPHRI Care and Public Health Research Institute (Maastricht, The Netherlands), NUTRIM School for Nutrition and Translational Research in Metabolism (Maastricht, the Netherlands), Stichting Annadal (Maastricht, The Netherlands), Health Foundation Limburg (Maastricht, The Netherlands), and by unrestricted grants from Janssen‐Cilag B.V. (Tilburg, The Netherlands), Novo Nordisk Farma B.V. (Alphen aan den Rijn, the Netherlands), and Sanofi‐Aventis Netherlands B.V. (Gouda, the Netherlands).

## CONFLICT OF INTEREST

None.

## Supporting information


Appendix S1

## Data Availability

The data that support the findings of this study are available on request from the corresponding author. The data are not publicly available due to privacy or ethical restrictions.
