# Supplementary material for: Daily patterns of physical activity, sedentary behavior, and prevalent and incident depression—The Maastricht Study
Source: Scand J Med Sci Sports. 2022 Sep 27;32(12):1768–80. doi: 10.1111/sms.14235 (PMC9827855; doi:10.1111/sms.14235)
Supplement: Supplementary file 1 — Appendix S1 [file SMS-32-1768-s001.docx]

**
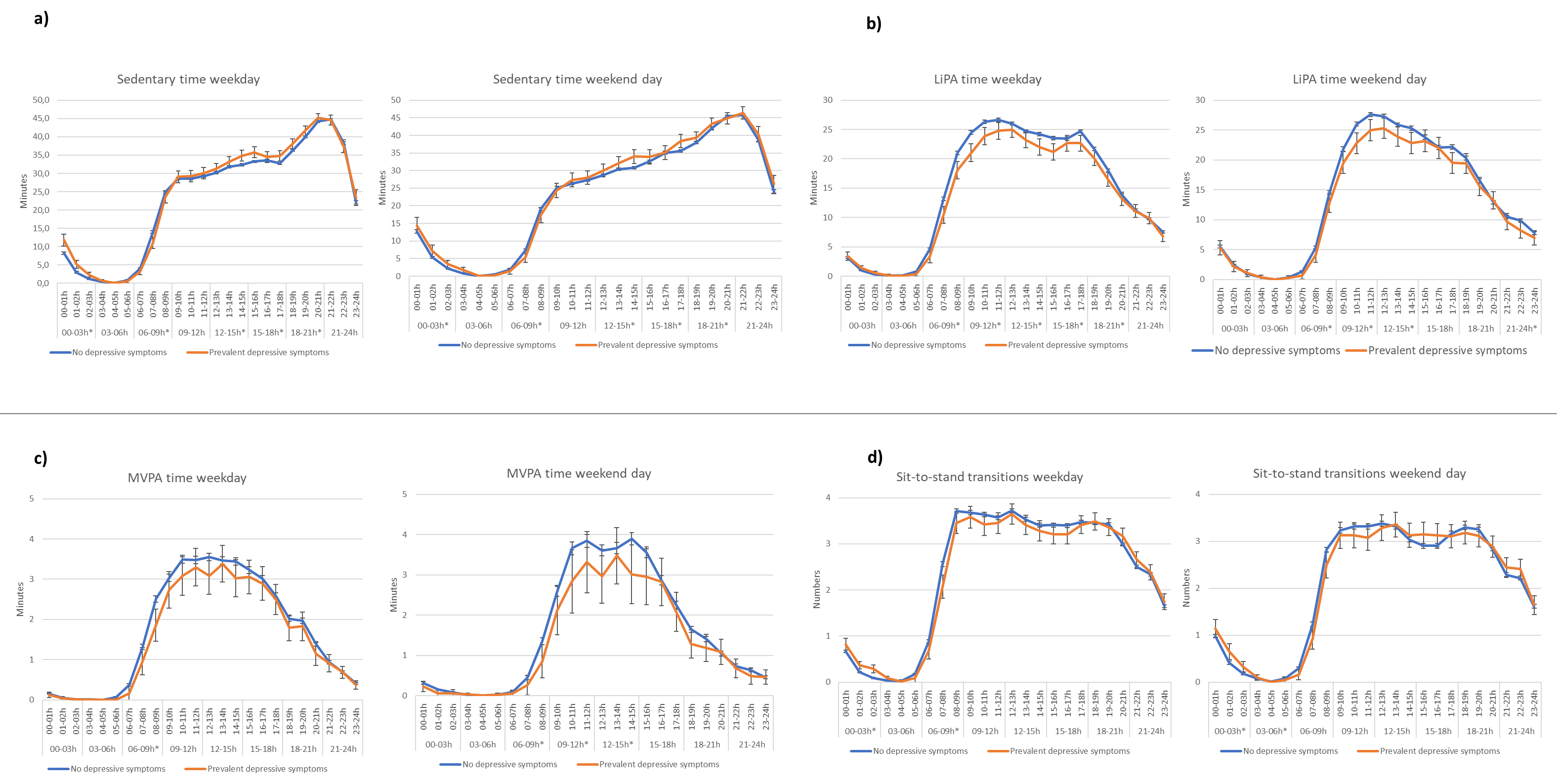
**

Statistically significant differences in time slots are reported with *. Model: adjusted for age, sex, level of education, type 2 diabetes, smoking status, alcohol consumption, energy intake, body mass index, hypertension, cholesterol, history of cardiovascular diseases.

**Supplementary Figure 1. Hourly distribution of a) sedentary time, b) light physical activity (LiPA), c) moderate-to-vigorous Physical activity (MVPA), and d) sit-to-stand transitions stratified by week and week-end days in individuals with and without prevalent depressive symptoms (n=**5,582**).**

**
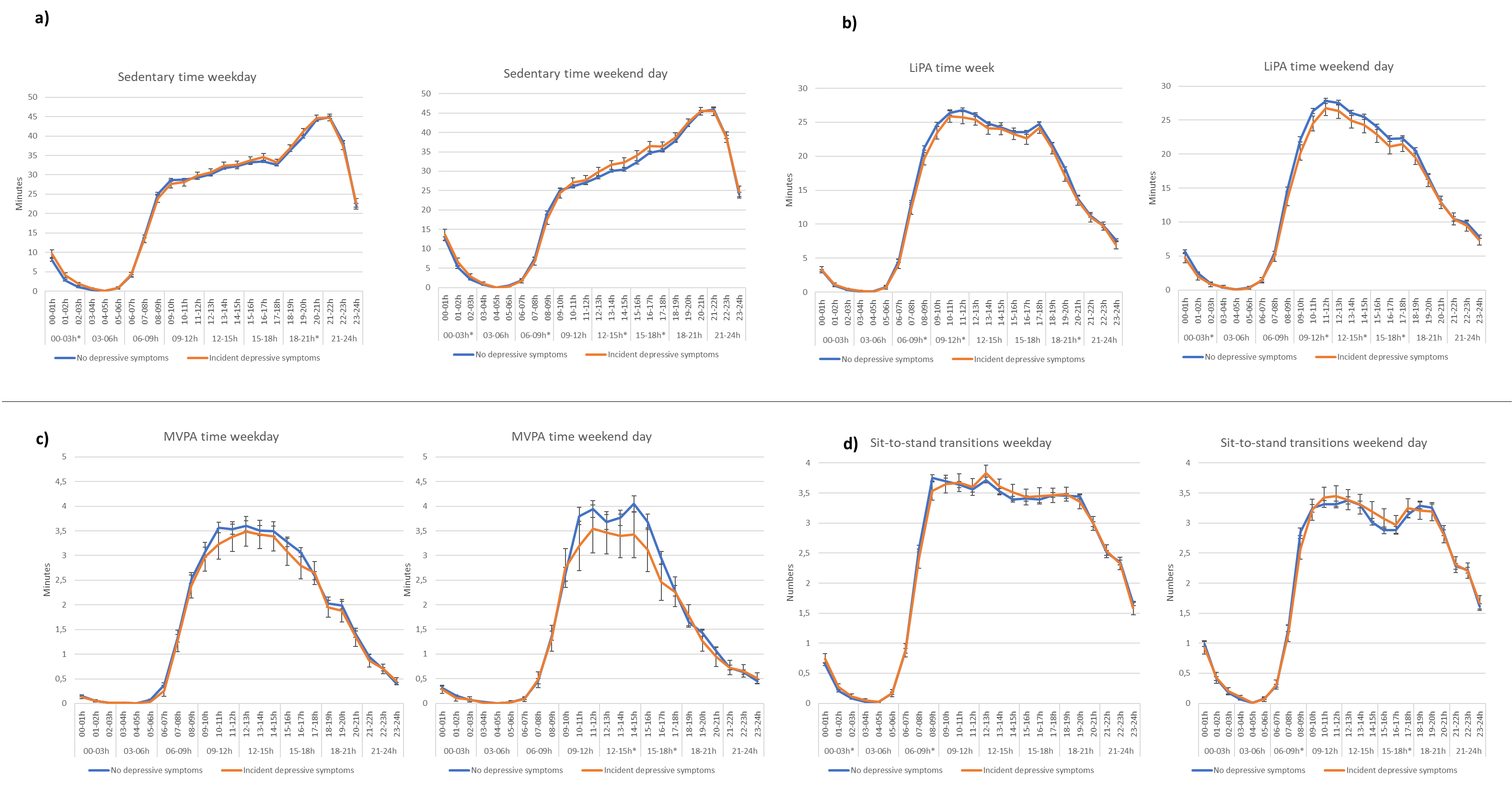
**Statistically significant differences in time slots are reported with *. Model: adjusted for age, sex, level of education, type 2 diabetes, smoking status, alcohol consumption, energy intake, body mass index, hypertension, cholesterol, history of cardiovascular diseases.

**Supplementary Figure 2. Hourly distribution of a) sedentary time, b) light physical activity (LiPA), c) moderate-to-vigorous physical activity (MVPA), and d) sit-to-stand transitions stratified by week and weekend days in individuals with and without incident depressive symptoms (n=5,113).**

**Supplementary Table 1 Characteristics of individuals with full data and with missing data in depressive symptoms (PHQ-9 at baseline) and missing data in physical activities and covariates for the cross-sectional analysis; and excluded due to depression at baseline and missing data in PHQ-9 during follow-up for the longitudinal analysis.**

|  | **Cross-sectional analysis** | | **Longitudinal analysis** | |
| --- | --- | --- | --- | --- |
| **Characteristic** | **Population with full data**  **(n=5,582)** | **Population with missing data**  **(n=2,107)** | **Population with full data**  **(n=5,113)** | **Population with missing data**  **(n=2,576)** |
| Sex (women), n (%) | 2,809 (50.3) | 1006 (47.7) | 2,559 (50.0) | 1,256 (48.8) |
| Age (years) | 59.9 ± 8.6 | 59.5 ± 8.8 | 60.1 ± 8.5 |  |
| Education, n (%)  *Low*  *Medium*  *High* | 1,891 (33.9)  1,537 (27.5)  2,154 (38.6) | 739 (37.1)  555 (27.9)  698 (35.0) | 1,682 (32.9)  1,408 (27.5)  2,023 (39.6) | 948 (38.5)  684 (27.8)  829 (33.7) |
| Smoking, n (%)  *Never*  *Former*  *Current* | 2,115 (37.9)  2,793 (50.0)  674 (12.1) | 720 (35.2)  971 (47.5)  352 (17.2) | 1,962 (38.4)  2,573 (50.3)  578 (11.3) | 873 (34.8)  1,191 (47.4)  448 (17.8) |
| Alcohol consumption, n (%)  *None*  *Low*  *High* | 981 (17.6)  3,304 (59.2)  1,297 (23.2) | 431 (21.1)  1,127 (55.2)  484 (23.7) | 835 (16.3)  3,052 (59.7)  1,226 (24.0) | 577 (23.0)  1,379 (54.9)  555 (22.1) |
| Having a partner (yes), n (%) | 4,707 (84.4) | 1,642 (80.3) | 4.364 (85.4) | 1,985 (78.9) |
| Energy intake (Kcal) | 2,128.3 ± 592.1 | 2,188.4 ± 631.4 | 2,126.4 ± 586.2 |  |
| BMI (kg/m^2^) | 26.9 ± 4.4 | 27.4 ± 4.8 | 26.8 ± 4.3 |  |
| Hypertension, n (%) | 2,989 (53.5) | 1,148 (54.8) | 2,721 (53.2) | 1,416 (55.2) |
| Total cholesterol-to-HDL cholesterol ratio, n (%) | 3.6 ± 1.2 | 3.7 ± 1.2 | 3.6 ± 1.1 |  |
| History of CVD, n (%) | 928 (16.6) | 370 (18.4) | 828 (16.2) | 470 (19.0) |
| Type 2 diabetes, n (%) | 1,298 (23.3) | 595 (28.2) | 1,136 (22.2) | 757 (29.4) |
| **Depression** | | |  |  |
| Depression score at baseline (PHQ-9 score) | 2.7 ± 3.3 | 3.1 ± 3.7 | 2.29 ± 2.3 |  |
| Major depressive disorder at baseline (MINI), n (%) | 152 (2.8) | 95 (4.8) | 68 (1.4) | 179 (7.3) |
| Major depressive disorder lifetime (MINI), n (%) | 1,635 (30.4) | 722 (36.3) | 1,381 (28.1) | 976 (39.9) |
| Use of antidepressants at baseline | 361 (6.5) | 190 (9.0) | 282 (5.5) | 269 (10.5) |
| **Physical activity** | | |  |  |
| Sedentary (minutes/day) | 561.1 ± 100.6 | 565.6 ± 114.6 | 559.9 ± 99.1 | 568.7 ± 115.0 |
| LiPA (minutes/day) | 343.5 ± 94.6 | 342.6 ± 104.3 | 344.9 ± 93.4 | 337.7 ± 105.3 |
| MVPA (minutes/day) | 40.1 ± 23.1 | 36.9 ± 26.6 | 40.7 ± 23.2 | 35.9 ± 24.9 |
| Transitions (n/day) | 54.6 ± 14.3 | 54.8 ± 15.3 | 54.6 ± 14.1 | 54.5 ± 15.6 |
| Results are presented as mean ± standard deviation (SD) or n (%).  LiPA= Light physical activity; MDD=Major Depressive Disorder; MINI= Mini-International Neuropsychiatric Interview; MVPA= Moderate-to-vigorous physical activity; PHQ-9= 9-item Patient Health Questionnaire | | | | |

**Supplementary Table 2. Hourly distribution of sedentary time, LiPA, MVPA and sit-to-stand transitions for an average day in participants with and without prevalent depressive symptoms (n=5,582)**

|  | Sedentary (minutes) | | | LiPA (minutes) | | | MVPA (minutes) | | | Sit-to-stand transitions (n) | | |
| --- | --- | --- | --- | --- | --- | --- | --- | --- | --- | --- | --- | --- |
|  | No prevalent depressive symptoms  mean (95% CI) | Prevalent depressive symptoms  mean (95% CI) | p-value | No prevalent depressive symptoms  mean (95% CI) | Prevalent depressive symptoms  mean (95% CI) | p-value | No prevalent depressive symptoms  mean (95% CI) | Prevalent depressive symptoms  mean (95% CI) | p-value | No prevalent depressive symptoms  mean (95% CI) | Prevalent depressive symptoms  mean (95% CI) | p-value |
| 00-03h | 14.8 (14.2-15.3) | 20.8 (17.9-23.7) | <0.001 | 5.8 (5.6-6.1) | 6.3 (5.1-7.5) | 0.478 | 0.3 (0.3-0.3) | 0.2 (0.1-0.3) | 0.138 | 1.2 (1.1-1.2) | 1.7 (1.4-1.9) | <0.001 |
| 03-06h | 1.2 (1.1-1.4) | 1.6 (1.0-2.1) | 0.255 | 0.9 (0.8-1.0) | 0.7 (0.2-1.1) | 0.265 | 0.1 (0.1-0.1) | 0.0 (0.0-0.1) | 0.115 | 0.2 (0.2-0.2) | 0.2 (0.1-0.3) | 0.708 |
| 06-09h | 38.5 (37.9-39.1) | 33.8 (30.8-36.8) | 0.003 | 33.4 (32.9-34.0) | 27.7 (25.0-30.4) | <0.001 | 3.5 (3.4-3.6) | 2.4 (1.8-3.0) | <0.001 | 6.3 (6.2-6.4) | 5.4 (5.0-5.9) | <0.001 |
| 09-12h | 84.1 (83.4-84.8) | 85.2 (81.7-88.7) | 0.536 | 76.9 (76.2-77.6) | 69.6 (66.2-73.0) | <0.001 | 10.0 (9.8-10.2) | 8.9 (7.8-9.9) | 0.040 | 10.6 (10.5-10.7) | 10.1 (9.6-10.6) | 0.080 |
| 12-15h | 93.0 (92.4-93.7) | 97.8 (94.5-101.1) | 0.005 | 76.0 (75.3-76.6) | 71.2 (68.1-74.3) | 0.003 | 10.7 (10.5-10.9) | 9.5 (8.5-10.6) | 0.031 | 10.4 (10.3-10.5) | 10.2 (9.7-10.6) | 0.447 |
| 15-18h | 100.6 (99.9-101.2) | 105.7 (102.4-109.0) | 0.003 | 70.7 (70.0-71.3) | 66.0 (63.0-69.1) | 0.004 | 8.8 (8.6-8.9) | 8.3 (7.4-9.1) | 0.266 | 9.9 (9.8-10.0) | 9.7 (9.3-10.2) | 0.502 |
| 18-21h | 121.9 (121.4-122.5) | 125.7 (122.8-128.7) | 0.013 | 52.4 (51.9-53.0) | 49.2 (46.5-52.0) | 0.027 | 5.0 (4.9-5.1) | 4.3 (3.7-4.9) | 0.028 | 9.7 (9.6-9.8) | 9.7 (9.4-10.1) | 0.907 |
| 21-24h | 106.3 (105.5-107.1) | 108.4 (104.4-112.4) | 0.311 | 28.4 (27.9-28.9) | 27.1 (24.8-29.4) | 0.280 | 2.0 (1.9-2.0) | 1.9 (1.5-2.2) | 0.506 | 6.4 (6.3-6.5) | 6.8 (6.4-7.1) | 0.047 |
| Total | 560.4 (557.9-562.9) | 579.0 (566.7-591.4) | 0.004 | 344.6 (342.1-347.0) | 317.8 (305.8-329.8) | <0.001 | 40.3 (39.7-40.9) | 35.5 (32.7-38.4) | 0.001 | 54.6 (54.2-55.0) | 53.8 (52.0-55.7) | 0.430 |
| Weekday | 562.7 (559.9-565.4) | 580.7 (567.2-594.2) | 0.010 | 350.4 (347.8-353.0) | 323.7 (310.8-336.7) | <0.001 | 41.1 (40.5-41.8) | 36.9 (33.9-39.9) | 0.007 | 56.5 (56.1-55.7) | 55.5 (53.5-57.5) | 0.323 |
| Weekend day | 555.7 (552.7-558.8) | 574.7 (559.7-589.7) | 0.015 | 330.9 (328.1-333.7) | 304.1 (290.3-318.0) | <0.001 | 38.3 (37.6-39.1) | 32.3 (28.6-36.0) | 0.002 | 50.2 (49.8-50.6) | 50.1 (48.1-52.1) | 0.892 |
| Model adjusted for: age, sex, level of education, type 2 diabetes, smoking status, alcohol consumption, energy intake, body mass index, hypertension, total cholesterol-to-HDL cholesterol ratio, history of cardiovascular diseases. LiPA= Light physical activity; MVPA= Moderate-to-vigorous physical activity | | | | | | | | | | | | |

**Supplementary Table 3. Hourly distribution of sedentary time, LiPA, MVPA and sit-to-stand transitions for an average day in participants with and without incident depressive symptoms (n=5,113)**

|  | Sedentary (minutes) | | | LiPA (minutes) | | | MVPA (minutes) | | | Sit-to-stand transitions (n) | | |
| --- | --- | --- | --- | --- | --- | --- | --- | --- | --- | --- | --- | --- |
|  | No prevalent depressive symptoms  mean (95% CI) | Incident depressive symptoms  mean (95% CI) | p-value | No prevalent depressive symptoms  mean (95% CI) | Incident depressive symptoms  mean (95% CI) | p-value | No prevalent depressive symptoms  mean (95% CI) | Incident depressive symptoms  mean (95% CI) | p-value | No prevalent depressive symptoms  mean (95% CI) | Incident depressive symptoms  mean (95% CI) | p-value |
| 00-03h | 14.2 (13.5-14.8) | 17.8 (16.1-19.6) | <0.001 | 5.8 (5.5-6.1) | 5.6 (4.8-6.4) | 0.657 | 0.3 (0.3-0.3) | 0.3 (0.2-0.3) | 0.392 | 1.1 (1.1-1.2) | 1.3 (1.1-1.4) | 0.081 |
| 03-06h | 1.2 (1.1-1.3) | 1.5 (1.2-1.8) | 0.077 | 0.9 (0.8-1.0) | 0.9 (0.6-1.2) | 0.981 | 0.1 (0.1-0.1) | 0.0 (0.0-0.1) | 0.20 | 0.2 (0.2-0.2) | 0.2 (0.2-0.3) | 0.400 |
| 06-09h | 38.9 (38.2-39.5) | 37.0 (35.1-38.9) | 0.064 | 33.9 (33.3-34.5) | 31.2 (29.5-32.9) | 0.003 | 3.5 (3.4-3.7) | 3.3 (2.9-3.7) | 0.255 | 6.4 (6.3-6.5) | 6.0 (5.7-6.3) | 0.013 |
| 09-12h | 84.0 (83.3-84.8) | 83.8 (81.6-86.0) | 0.842 | 77.4 (76.7-78.2) | 74.0 (71.9-76.0) | 0.002 | 10.2 (10.0-10.5) | 9.6 (8.9-10.3) | 0.084 | 10.6 (10.5-10.7) | 10.7 (10.4-11.0) | 0.581 |
| 12-15h | 92.6 (91.9-93.3) | 94.9 (92.9-100.9) | 0.035 | 76.3 (75.6-76.9) | 74.1 (72.3-76.0) | 0.037 | 10.9 (10.6-11.1) | 10.3 (9.7-10.9) | 0.094 | 10.4 (10.3-10.5) | 10.6 (10.3-10.9) | 0.108 |
| 15-18h | 100.2 (99.5-100.9) | 103.0 (101.0-105.1) | 0.010 | 70.9 (70.2-71.6) | 68.7 (66.8-70.6) | 0.033 | 8.9 (8.7-9.1) | 8.3 (7.7-8.8) | 0.021 | 9.9 (98-10.0) | 10.0 (9.8-10.3) | 0.228 |
| 18-21h | 121.6 (121.0-122.3) | 123.7 (121.9-125.5) | 0.035 | 52.7 (52.1-53.3) | 50.8 (49.1-52.5) | 0.044 | 5.0 (4.9-5.2) | 4.8 (4.5-5.2) | 0.335 | 9.7 (9.7-9.8) | 9.6 (9.4-9.9) | 0.391 |
| 21-24h | 106.3 (105.4-107.1) | 106.0 (103.6-107.1) | 0.871 | 28.5 (28.0-29.0) | 27.6 (26.1-29.0) | 0.237 | 2.0 (1.9-2.1) | 2.0 (1.8-2.2) | 0.980 | 6.4 (6.3-6.5) | 6.3 (6.1-6.6) | 0.665 |
| Total | 559.1 (556.4-561.8) | 567.3 (559.7-575.0) | 0.046 | 346.3 (343.7-348.9) | 332.8 (325.3-340.2) | 0.001 | 40.9 (40.3-41.5) | 38.6 (36.8-40.4) | 0.016 | 54.6 (54.2-55.0) | 54.7 (53.6-55.8) | 0.840 |
| Weekday | 562.0 (559.0-564.9) | 568.3 (559.9-576.6) | 0.164 | 351.7 (348.8-354.5) | 339.8 (331.7-347.8) | 0.003 | 41.7 (41.0-42.3) | 39.8 (37.9-41.6) | 0.062 | 56.5 (56.1-57.0) | 56.6 (55.3-57.8) | 0.945 |
| Weekend day | 553.2 (549.9-556.5) | 565.4 (556.1-574.8) | 0.015 | 33.6 (330.6-336.6) | 316.4 (307.8-325.1) | <0.001 | 39.2 (38.4-40.0) | 35.9 (33.6-38.2) | 0.009 | 50.1 (49.7-50.6) | 50.5 (49.3-51.7) | 0.558 |
| Model adjusted for: age, sex, level of education, type 2 diabetes, smoking status, alcohol consumption, energy intake, body mass index, hypertension, total cholesterol-to-HDL cholesterol ratio, history of cardiovascular diseases. LiPA= Light physical activity; MVPA= Moderate-to-vigorous physical activity | | | | | | | | | | | | |

**Supplementary Table 4. Hourly distribution of sedentary, LiPA, MVPA and sit-to-stand transitions for an average day in participants with and without incident depressive symptoms; sensitivity analysis excluding individuals with MDD at baseline (n= 4,860).**

|  | Excluded individuals with MDD at baseline | | | | | | | | | | | |
| --- | --- | --- | --- | --- | --- | --- | --- | --- | --- | --- | --- | --- |
|  | Sedentary (minutes) | | | LiPA (minutes) | | | MVPA (minutes) | | | Sit-to-stand transitions (n) | | |
|  | No prevalent depressive symptoms  mean (95% CI) | Incident depressive symptoms  mean (95% CI) | p-value | No prevalent depressive symptoms  mean (95% CI) | Incident depressive symptoms  mean (95% CI) | p-value | No prevalent depressive symptoms  mean (95% CI) | Incident depressive symptoms  mean (95% CI) | p-value | No prevalent depressive symptoms  mean (95% CI) | Incident depressive symptoms  mean (95% CI) | p-value |
| 00-03h | 14.3 (13.6-14.9) | 17.2 (15.4-19.1) | 0.003 | 5.8 (5.6-6.1) | 55. (4.7-6.3) | 0.493 | 0.3 (0.3-0.3) | 0.3 (0.2-0.3) | 0.171 | 1.1 (1.1-1.2) | 1.2 (1.1-1.4) | 0.251 |
| 03-06h | 1.2 (1.0-1.3) | 1.4 (1.1-1.7) | 0.152 | 0.9 (0.8-1.0) | 0.9 (0.6-1.2) | 0.9847 | 0.1 (0.1-0.1) | 0.0 (0.0-0.1) | 0.189 | 0.2 (0.2-0.2) | 0.2 (0.2-0.3) | 0.627 |
| 06-09h | 38.7 (38.0-39.3) | 37.1 (35.1-39.0) | 0.132 | 33.6 (33.0-34.2) | 31.2 (29.5-33.0) | 0.011 | 3.5 (3.4-3.6) | 3.3 (2.9-3.7) | 0.313 | 6.3 (6.2-6.4) | 6.0 (5.7-6.3) | 0.029 |
| 09-12h | 83.9 (83.1-84.7) | 83.9 (81.6 (86.2) | 0.982 | 77.4 (76.7-78.2) | 74.0 (71.8-76.2) | 0.005 | 10.3 (10.0-10.5) | 9.6 (8.9-10.3) | 0.088 | 10.6 (10.5-10.7) | 10.7 (10.4-11.1) | 0.391 |
| 12-15h | 92.4 (91.7-93.2) | 95.5 (93.4-97.6) | 0.007 | 76.4 (75.7-77.0) | 73.6 (71.6-75.5) | 0.009 | 10.9 (10.7-11.2) | 10.3 (9.7-11.0) | 0.090 | 10.4 (10.3-10.5) | 10.6 (10.3-10.9) | 0.160 |
| 15-18h | 100.1 (99.3-100.8) | 103.0 (100.9-105.2) | 0.011 | 70.9 (70.2-71.6) | 68.7 (66.7-70.7) | 0.039 | 9.0 (8.8-9.2) | 8.3 (7.7-8.8) | 0.017 | 9.9 (9.8-10.0) | 10.1 (9.8-10.3) | 0.193 |
| 18-21h | 121.6 (121.0-122.3) | 123.8 (121.9-125.7) | 0.038 | 52.7 (52.1-53.3) | 50.7 (48.9-52.5) | 0.039 | 5.1 (4.9-5.2) | 4.9 (4.5-5.3) | 0.316 | 9.7 (9.6-9.8) | 9.7 (9.4-9.9) | 0.645 |
| 21-24h | 106.8 (105.9-107.7) | 105.7 (103.1-108.3) | 0.436 | 28.5 (28.0-29.0) | 27.5 (26.0-29.0) | 0.211 | 2.0 (1.9-2.1) | 2.0 (1.7-2.2) | 0.797 | 6.4 (6.3-6.5) | 6.3 (6.1-6.6) | 0.600 |
| Total | 558.9 (556.2-561.7) | 567.6 (559.6-575.6) | 0.045 | 346.2 (343.6-348.9) | 332.1 (324.3-339.8) | 0.001 | 41.2 (40.5-41.8) | 38.6 (36.8-40.5) | 0.013 | 54.6 (54.2-55.1) | 54.9 (53.7-56.1) | 0.737 |
| Weekday | 561.9 (558.9-564.9) | 569.0 (560.2-577.7) | 0.134 | 351.6 (348.7-354.5) | 338.9 (330.5-347.3) | 0.005 | 41.9 (41.2-42.6) | 39.8 (37.9-41.8) | 0.052 | 56.6 (56.2-57.1) | 56.8 (55.5-58.1) | 0.838 |
| Weekend day | 552.8 (549.5-556.2) | 564.5 (554.8-574.2) | 0.026 | 333.6 (330.5-336.7) | 316.4 (307.4-325.3) | <0.001 | 39.5 (38.6-40.3) | 36.0 (33.5-38.4) | 0.008 | 50.1 (49.7-50.6) | 50.6 (49.3-51.9) | 0.509 |
| Model adjusted: for age, sex, level of education, type 2 diabetes, smoking status, alcohol consumption, energy intake, body mass index, hypertension, total cholesterol-to-HDL cholesterol ratio, history of cardiovascular diseases. LiPA= Light physical activity; MDD=Major Depressive Disorder; MVPA= Moderate-to-vigorous physical activity | | | | | | | | | | | | |

**Supplementary Table 5. Hourly distribution of sedentary, LiPA, MVPA and sit-to-stand transitions for an average day in participants with and without incident depressive symptoms; sensitivity analysis excluding individuals with antidepressant drugs use at baseline (n= 4,831).**

|  | Excluded individuals with antidepressant drugs use at baseline | | | | | | | | | | | |
| --- | --- | --- | --- | --- | --- | --- | --- | --- | --- | --- | --- | --- |
|  | Sedentary (minutes) | | | LiPA (minutes) | | | MVPA (minutes) | | | Sit-to-stand transitions (n) | | |
|  | No prevalent depressive symptoms  mean (95% CI) | Incident depressive symptoms  mean (95% CI) | p-value | No prevalent depressive symptoms  mean (95% CI) | Incident depressive symptoms  mean (95% CI) | p-value | No prevalent depressive symptoms  mean (95% CI) | Incident depressive symptoms  mean (95% CI) | p-value | No prevalent depressive symptoms  mean (95% CI) | Incident depressive symptoms  mean (95% CI) | p-value |
| 00-03h | 14.4 (13.7-15.0) | 16.9 (15.0-18.8) | 0.013 | 5.9 (5.6-6.1) | 5.6 (4.8-6.5) | 0.599 | 0.3 (0.3-0.3) | 0.3 (0.2-0.4) | 0.477 | 1.1 (1.1-1.2) | 1.2 (1.1-1.4) | 0.270 |
| 03-06h | 1.2 (1.1-1.3) | 1.5 (1.2-1.9) | 0.048 | 0.9 (0.8-1.0) | 1.0 (0.7-1.3) | 0.789 | 0.1 (0.1-0.1) | 0.0 (0.0-0.1) | 0.277 | 0.2 (0.2-0.2) | 0.2 (0.2-0.3) | 0.186 |
| 06-09h | 39.1 (38.4-39.8) | 38.6 (36.6-40.6) | 0.668 | 34.0 (33.4-34.6) | 32.9 (31.1-34.7) | 0.251 | 3.6 (3.4-3.7) | 3.5 (3.1-3.9) | 0.943 | 6.4 (6.3-6.5) | 6.3 (6.0-6.6) | 0.392 |
| 09-12h | 84.0 (83.2-84.8) | 83.7 (81.4-86.0) | 0.809 | 77.6 (76.9-78.4) | 75.5 (73.3-77.8) | 0.088 | 10.3 (10.0-10.5) | 9.9 (9.2-10.6) | 0.295 | 10.6 (10.5-10.7) | 10.8 (10.5-11.1) | 0.291 |
| 12-15h | 92.5 (91.7-93.2) | 94.2 (92.1-96.4) | 0.134 | 76.4 (75.7-77.0) | 74.7 (72.6-76.7) | 0.120 | 10.9 (10.7-11.1) | 10.6 (9.9-11.3) | 0.399 | 10.4 (10.3-10.5) | 10.6 (10.3-10.9) | 0.086 |
| 15-18h | 100.0 (99.3-100.8) | 101.9 (99.7-104.1) | 0.124 | 71.0 (70.3-71.7) | 69.6 (67.6-71.7) | 0.212 | 9.0 (8.8-9.1) | 8.5 (7.9-9.1) | 0.174 | 9.8 (9.7-9.9) | 10.1 (9.8-10.4) | 0.128 |
| 18-21h | 121.5 (120.8-122.1) | 123.1 (121.2-125.1) | 0.115 | 52.8 (52.2-53.5) | 51.2 (49.3-53.0) | 0.094 | 5.1 (5.0-5.2) | 5.0 (4.6-5.5) | 0.828 | 9.7 (9.6-9.8) | 9.7 (9.5-10.0) | 0.785 |
| 21-24h | 106.6 (105.7-107.5) | 106.2 (103.6-108.9) | 0.812 | 28.7 (28.1-29.2) | 27.7 (26.2-29.3) | 0.266 | 2.0 (1.9-2.1) | 2.1 (1.8-2.3) | 0.597 | 6.4 (6.3-6.5) | 6.4 (6.1-6.6) | 0.738 |
| Total | 559.2 (556.5-561.9) | 566.3 (558.1-574.5) | 0.109 | 347.3 (344.6-350.0) | 338.2 (330.3-346.2) | 0.035 | 41.2 (40.5-41.8) | 40.0 (38.0-41.9) | 0.252 | 54.6 (54.2-55.0) | 55.3 (54.1-56.5) | 0.314 |
| Weekday | 562.2 (559.2-565.1) | 566.8 (557.9-575.8) | 0.333 | 352.7 (349.9-355.6) | 345.9 (337.3-354.5) | 0.143 | 41.9 (41.2-42.6) | 41.1 (39.0-43.1) | 0.476 | 56.6 (56.1-57.0) | 57.1 (55.8-58.4) | 0.458 |
| Weekend day | 553.2 (549.9-556.5) | 564.8 (554.8-574.8) | 0.031 | 334.5 (331.5-337.6) | 320.5 (331.3-329.7) | 0.005 | 39.5 (38.7-40.4) | 37.3 (34.8-39.9) | 0.112 | 50.2 (49.7-50.6) | 51.1 (49.8-52.4) | 0.175 |
| Model adjusted: for age, sex, level of education, type 2 diabetes, smoking status, alcohol consumption, energy intake, body mass index, hypertension, total cholesterol-to-HDL cholesterol ratio, history of cardiovascular diseases. LiPA= Light physical activity; MVPA= Moderate-to-vigorous physical activity | | | | | | | | | | | | |
